# Thrombotic prosthetic valve dysfunction requiring valve re-replacement after mitral valve replacement for caseous calcification with hypereosinophilic syndrome: a case report

**DOI:** 10.1093/ehjcr/ytaf024

**Published:** 2025-01-22

**Authors:** Keishi Miyazawa, Eriya Imai, Ami Kodaira, Toshiki Ota, Motoi Yokozuka

**Affiliations:** Department of Anesthesia, Saiseikai Yokohamashi Tobu Hospital, 3-6-1 Shimosueyoshi, Tsurumi-ku, Yokohama, Kanagawa 230-0012, Japan; Division of Anesthesia, Mitsui Memorial Hospital, 1 Kandaizumicho, Chiyoda-ku, Tokyo 101-8643, Japan; Division of Anesthesia, Mitsui Memorial Hospital, 1 Kandaizumicho, Chiyoda-ku, Tokyo 101-8643, Japan; Division of Anesthesia, Mitsui Memorial Hospital, 1 Kandaizumicho, Chiyoda-ku, Tokyo 101-8643, Japan; Division of Anesthesia, Mitsui Memorial Hospital, 1 Kandaizumicho, Chiyoda-ku, Tokyo 101-8643, Japan; Division of Anesthesia, Mitsui Memorial Hospital, 1 Kandaizumicho, Chiyoda-ku, Tokyo 101-8643, Japan

**Keywords:** Caseous calcification of the mitral annulus, Mitral annulus calcification, Prosthetic valve dysfunction, Hypereosinophilic syndrome, Eosinophilia, Calcinosis, Case report

## Abstract

**Background:**

Caseous calcification of the mitral annulus (CCMA) and hypereosinophilic syndrome (HES) are both associated with thrombotic tendencies. Caseous calcification of the mitral annulus may cause mitral valve regurgitation (MR), which may necessitate surgical intervention, depending on its severity. Given the absence of reported cases combining CCMA and HES, the optimal target range for anticoagulation therapy after mitral valve replacement remains to be established.

**Case summary:**

A 64-year-old man with CCMA complicated by HES was admitted due to heart failure and severe MR. The patient underwent mitral valve replacement with a mechanical valve. Despite standard anticoagulation therapy, prosthetic valve dysfunction had developed because of thrombosis. Intraoperative transoesophageal echocardiography revealed a closed stuck valve, necessitating valve re-replacement. A bioprosthetic valve was selected.

**Discussion:**

In cases of CCMA complicated by HES, the target of anticoagulation therapy is unclear. This case demonstrated that more intensive anticoagulation is required because of the thrombogenic risk of CCMA and HES. The mechanism of calcification due to eosinophilia has been suggested, and the relationship between eosinophilia and CCMA remains a subject for future research.

**Learning points**

- Caseous calcification of the mitral annulus (CCMA) and hypereosinophilic syndrome are both associated with thrombotic tendencies. A bioprosthetic valve may be selected and careful consideration of anticoagulation therapy is required after valve replacement surgery.
- Eosinophilia may contribute to the development of CCMA.

## Introduction

Caseous calcification of the mitral annulus (CCMA) is a subtype of mitral annular calcification^1^ with a prevalence of ∼0.06%.^2^ Caseous calcification of the mitral annulus can alter blood flow through the mitral valve and cause mitral valve regurgitation (MR).^3^ Cardioembolic stroke can occur in 19.2% of CCMA patient.^4^ Surgical intervention is indicated for valvular dysfunction or cerebrovascular events.^3^

Hypereosinophilic syndrome (HES) is defined as unexplained and sustained eosinophilia lasting for 6 months with organ damage. The prevalence of HES is 3%,^5^ of which 40% patients develop eosinophilic myocarditis (EM).^6^

There have been no reports of CCMA coexisting with HES, nor have there been any cases of a thrombus-induced stuck valve after surgical mitral valve replacement (MVR), necessitating re-replacement.^3,5^

We report the case of a patient who underwent mechanical MVR for severe MR of CCMA and subsequently developed a closed stuck valve due to thrombosis and pannus, requiring re-replacement. The thrombus was likely attributable to a prothrombotic state induced by CCMA and HES.

## Summary figure

**Table:** 

Before admission	Eosinophilia was revealed at age 50. Caseous calcification of the mitral annulus (CCMA) was diagnosed at age 52.
At admission	Worsened severe mitral valve regurgitation needed surgical mitral valve replacement (MVR) with a mechanical valve at age 64.
First operation	MVR with a mechanical valve was performed.
Postoperative course	Warfarin was initiated with a prothrombin time–international normalized ratio (PT-INR) target of 1.6–2.0.
3 months after the surgery	The patient presented with exertional dyspnoea. Hypereosinophilic syndrome (HES) was diagnosed and pulse steroid therapy was administered. Intracardiac thrombus, paravalvular leakage, and structural valve dysfunction were revealed.
Second operation	Thrombus removal and paraleak closure was performed. Transoesophageal echocardiography showed the mechanical valve disc was stuck, which necessitated valve re-replacement with a bioprosthetic valve.
Postoperative course	Warfarin was continued with a PT-INR target of 2.0–2.6 despite a bioprosthetic valve because of the thrombogenic risk of CCMA and HES. No further thrombus formation was observed.

## Case description

**Figure 1:**
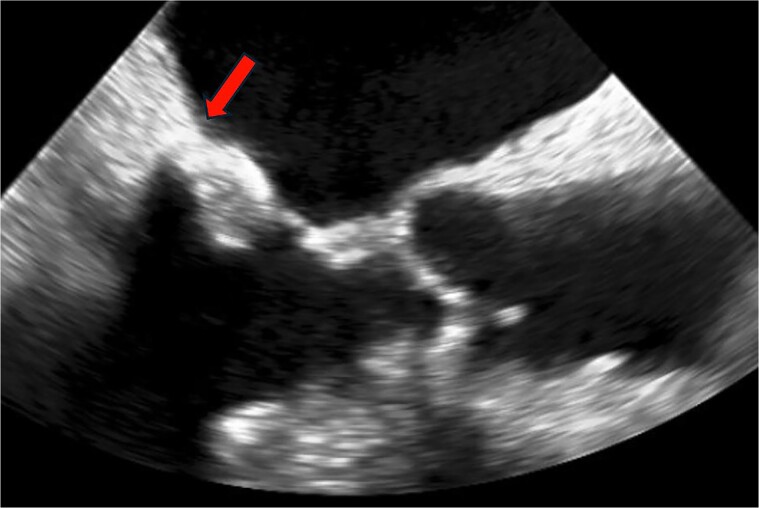
Transoesophageal echocardiography imaging of caseous calcification of the mitral annulus. The arrow indicates the mass-like appearance of caseous calcification of the mitral annulus.

The patient was a 64-year-old male with atrial fibrillation, lacunar infarction, bronchial asthma, and end-stage renal disease due to IgA nephropathy, requiring haemodialysis since age 32. At 50 years of age, blood tests revealed eosinophilia of undetermined aetiology, which fluctuated without intervention and consistently showed elevated levels. Intermittently, eosinophil counts exceeded 5000/μL. At 52 years of age, transoesophageal echocardiography (TEE) showed CCMA and MR due to calcification of P3 scallop of the posterior leaflet (*Figure 1*). As MR worsened over time, MVR was performed. A mechanical valve was selected as the standard surgical procedure in a specific guideline for patients 65 years of age or younger.^7^

Transthoracic echocardiography (TTE) revealed left ventricular end-diastolic/systolic diameter 49/30 mm and ejection fraction of 68%. He had severe MR due to the lack of coaptation from annular calcification (regurgitant volume 93 mL and effective regurgitant orifice area 0.62 cm^2^ by the proximal isovelocity surface area method).

**Figure 2:**
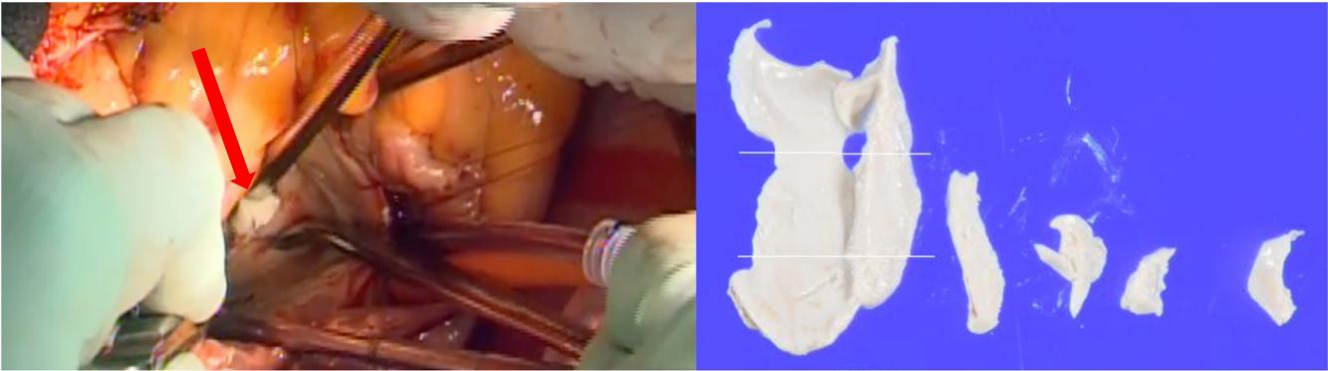
Surgical image (left) and surgical specimen of caseous calcification of the mitral annulus (right). Left: The arrow indicates a cement-like substance of the calcified lesion. Right: Histologically degenerative material with calcification is observed.

**Figure 3:**
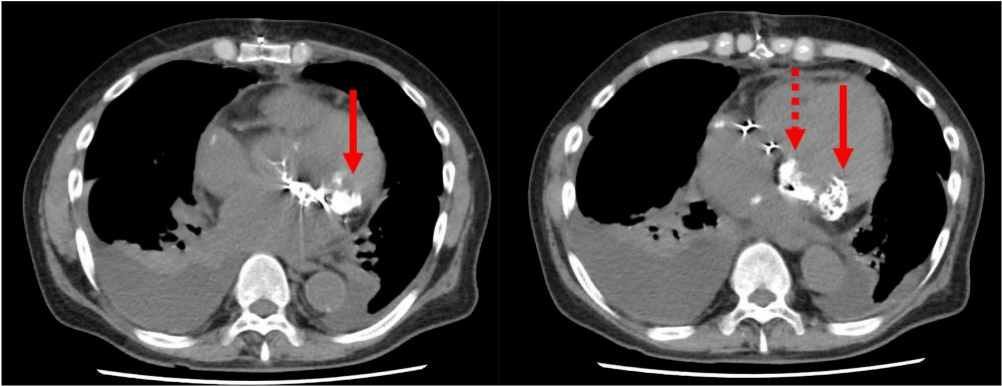
Computed tomography images after the first surgery. Left: Residual calcification (arrow) is visible outside the prosthetic valve. Right: Residual calcification is observed lateral (solid arrow) and posterior caudal (dashed arrow) to the prosthetic valve.

Surgical findings revealed that the posterior leaflet had a calcified lesion on the ventricular side; when this area was excised, a cement-like substance was observed extruding from within the calcification (*Figure 2*). The calcified area was excised, with efforts made to limit the extent of resection as much as feasible to prevent heart rupture. The mitral annulus was repaired with pledgeted sutures, and additional autologous pericardial pledgets were used to reinforce the severely calcified P3 region. A mechanical valve (29 mm, St. Jude Medical™ Masters Series Mechanical Heart Valve) was implanted. The P2 and P3 were reinforced by continuous suturing of the left atrial wall to the sewing cuff of the valve. Calcification lateral and posterior caudal to the mitral valve were intentionally left in place to prevent heart rupture (*Figure 3*). Warfarin (4 mg) was initiated on postoperative Day 4 with a prothrombin time–international normalized ratio (PT-INR) target of 1.6–2.0, adjusted to 1.5 mg. Aspirin was added on postoperative Day 6. Pathological findings of the mitral annulus confirmed CCMA.

**Figure 4:**
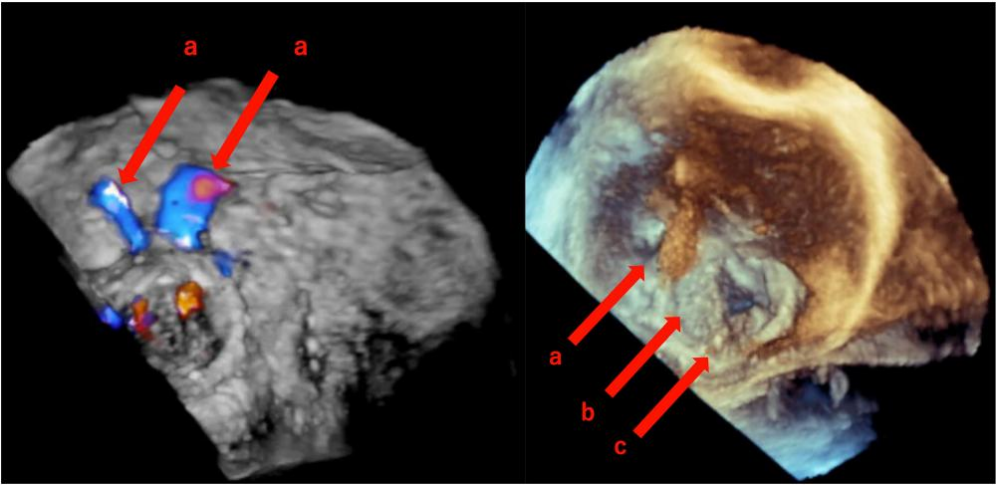
3D transoesophageal echocardiography. (*A*) Para-valvular leakage. (*B*) Closed stuck valve. (*C*) Thrombus.

**Figure 5:**
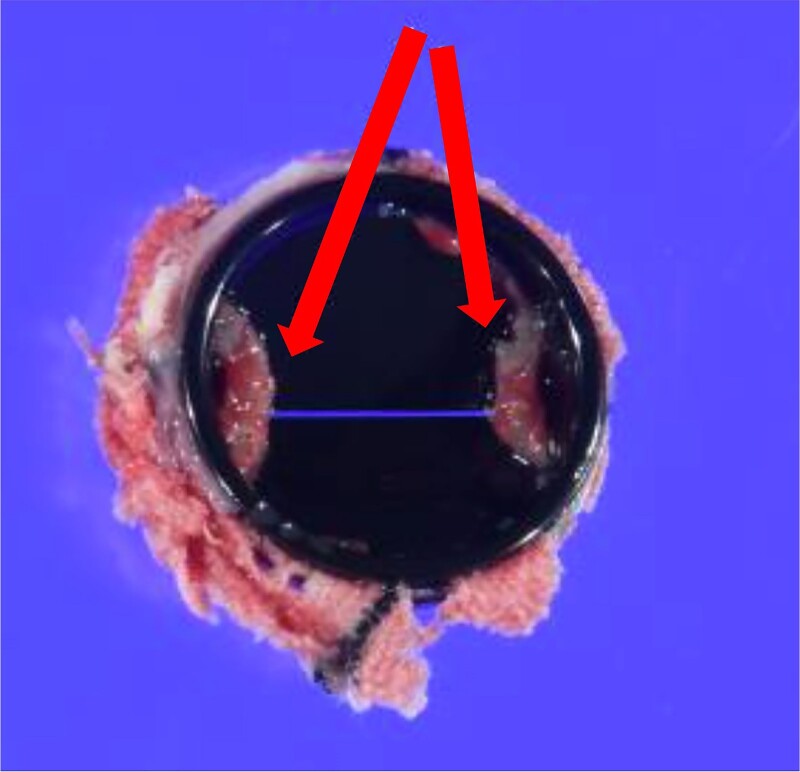
Explanted prosthetic valve. An organized fibrin thrombus (arrow) and pannus formation on both hinges on the ventricular side are observed.

Three months after surgery, the patient was admitted with exertional dyspnoea. The vital signs were as follows: blood pressure, 134/88 (103) mmHg; pulse rate, 99 b.p.m.; respiratory rate, 26/min; and SpO_2_, 96% (room air). Laboratory findings revealed anaemia (Hb, 7.6 g/dL), eosinophilia (7700/μL), and elevated LDH and indirect bilirubin levels. As the eosinophil count remained >5000/μL for over 6 months, along with intracardiac thrombus, the patient was diagnosed with HES. Pulse steroid therapy was administered for HES. Transthoracic echocardiography revealed new paravalvular leakage (PVL) and structural valve dysfunction causing haemolytic anaemia. The PVL originated from the side opposite to the calcified annulus excised during the surgery. No transvalvular leakage was observed. As cinefluoroscopy revealed worsened leaflet motion during opening and closing, TTE showed moderate mitral valve stenosis (mean pressure gradient, 10 mmHg), and surgery was urgently planned. A 7 × 4 × 1.5 cm thrombus was removed from the left atrial posterior wall. The old thrombi adhered to the prosthetic valve. The planned procedure involved thrombus removal and paraleak closure with a bovine pericardial patch. It was unclear whether a leaflet was stuck from the surgeon’s view, and the TEE evaluation before the cardioplegia showed that the mechanical valve disc was stuck closed (*Figure 4*); therefore, thrombus removal alone would be insufficient, which necessitated valve re-replacement. Pannus formation was observed on both hinges on the ventricular side of the explanted prosthetic valve (*Figure 5*). Considering the high risk of thrombogenesis with a mechanical valve owing to CCMA- and HES-induced hypercoagulability, a bioprosthetic valve (27 mm Mitris RESILIA, Edwards Lifesciences) was implanted.

Warfarin was initiated on postoperative Day 4 with a PT-INR target of 2.0–2.6, adjusting to 3 mg. The steroid dose was gradually tapered from 500 to 5 mg for HES; however, a low-dose steroid (5 mg) was required because of eosinophilia rebound. Warfarin was continued because of the thrombogenic risk of CCMA and HES. No further thrombus formation was observed.

## Discussion

In this rare case of CCMA complicated by HES, the patient developed prosthetic valve thrombosis despite receiving standard anticoagulant therapy after MVR. Eosinophilia may have contributed to the development of CCMA.

Prosthetic valve thrombosis occurred in this case of CCMA with HES, despite receiving standard anticoagulant therapy. In the first MVR, a mechanical valve was selected based on the patient’s age.^7^ The recommended anticoagulation target for warfarin in mechanical mitral valves is a PT-INR of 2.0–3.0.^7^ However, for dialysis patients, a lower target of 1.6–2.0 is recommended because of a higher risk of bleeding and a lower risk of embolism compared with Westerners’.^8^ After re-replacement with a bioprosthetic valve, anticoagulation therapy with warfarin, targeting a higher PT-INR of 2.0–2.6, was continued, preventing thrombus formation.

As there are no case reports of combined CCMA and HES, the optimal PT-INR target remains unclear. Both conditions predispose patients to thrombosis, making anticoagulation bridging with heparin until the target PT-INR is achieved with warfarin potentially beneficial.^9^ In cases complicated by HES, the thrombotic tendency is higher owing to the pathophysiology of HES-associated EM.^10^ Eosinophilic granules contain substances that promote coagulation and increase the risk of thrombosis.^11^ Caseous calcification of the mitral annulus is associated with thrombotic risk, which is attributed to turbulent blood flow around the calcified annulus and debris from the necrotic calcified septum.^3^ Thrombosis was attributed to CCMA and HES in the post-valve replacement state. Continued steroid administration was necessary to control HES,^12^ and a bioprosthetic valve may be selected.

Eosinophilia may have contributed to the development of CCMA in this case. The relationship between calcification and eosinophilia has been studied in coronary arteries, showing a positive correlation between eosinophils and coronary artery calcification.^13^ Eosinophilia is known to positively correlate not only with vascular calcification but also with mitral and aortic valve calcification. Blood eosinophils and eosinophil cationic protein (ECP) concentration are considered risk factors for cardiovascular disease.^14^ Eosinophil cationic protein released by eosinophils binds to receptors on vascular smooth muscle cells, promoting vascular calcification. Furthermore, eosinophil counts and ECP levels show positive correlations with mitral valve calcification scores.^14^ In this case, eosinophilia was noted prior to the first surgery, suggesting a possible contribution to CCMA. Future elucidation of the relationship among eosinophilia, ECP, and mitral valve calcification may advance understanding of CCMA pathogenesis.

Both HES and CCMA are known to induce thrombotic tendencies. Therefore, pre-emptive measures against eosinophilia and the selection of bioprosthetic valves less prone to thrombotic complications during valve replacement are necessary, in addition to perioperative anticoagulation therapy.

## Data Availability

Data supporting the findings of this study are available from the corresponding author upon reasonable request.

## Lead author biography

Keishi Miyazawa, MD, is an anaesthesia resident at Saiseikai Yokohamashi Tobu Hospital. He graduated from Tohoku University and completed his junior residency at Mitsui Memorial Hospital. He has a special interest in perioperative haemodynamics.

## Notes

**Consent:** The authors confirm that written consent for submission and publication of this case report including images and associated text has been obtained from the patient in line with COPE guidance.

**Funding**: This research received no specific grant from any funding agency in the public, commercial, or not-for-profit sectors.
